# Morphological, Structural, and Thermal Properties of Microbial Carotenoids Microencapsulated by Spray‐Drying Using Different Combinations of Wall Materials

**DOI:** 10.1111/1750-3841.71034

**Published:** 2026-04-06

**Authors:** Patrícia Griep, Rosicler Colet, Elton Franceschi, Clarice Steffens, Jamile Zeni, Eunice Valduga

**Affiliations:** ^1^ Department of Food Engineering URI Erechim Erechim Brazil; ^2^ Center for Research on Colloidal Systems (NUESC), Institute of Research and Technology (ITP) Tiradentes University (UNIT) Aracaju Brazil

## Abstract

This study evaluated the spray‐drying encapsulation of carotenoid‐rich extracts produced by *Sporidiobolus salmonicolor*, with the optimization of a ternary wall system of gum arabic (GA), inulin (IN), and corn starch (ST). Carotenoids were produced in a semi‐continuous bioreactor, extracted with organic solvents, and microencapsulated by spray drying. A 2^3^ CCRD was used to optimize the proportions of the wall materials. The microcapsules were subjected to physicochemical, morphological, structural, and thermal analyses. Optimal conditions (20% extract, 2% Tween 80, 5% wall material at a 1:1:1 ratio, 120°C inlet temperature, and 5.83 mL/min feed rate) achieved 75% EE, 20.5 µg/g carotenoid loading, 83% EY, 5.5% moisture, and 0.126 a*_w_*. SEM showed spherical, smooth particles, while XRD indicated an amorphous structure stable up to 215°C. The optimized ternary system produced stable microcapsules suitable for use as sustainable natural colorants for food and pharmaceutical applications.

**Practical Applications:**

The carotenoid microcapsules obtained from *Sporidiobolus salmonicolor* by spray drying using gum arabic, inulin, and starch show potential application as natural colorants and functional ingredients in food and pharmaceutical products. The observed technological properties, such as high encapsulation efficiency, low moisture content and water activity, an amorphous structure, and thermal stability, favor handling, stability, and incorporation into different matrices, including beverages, dairy products, and powdered foods. The developed system represents a sustainable alternative to synthetic pigments and a promising platform for the industrial utilization of microbial carotenoids.

## 1 Introduction

Food color, a fundamental sensory attribute, plays a crucial role in consumer perception and emotional responses, directly affecting product acceptance. The increasing awareness of issues related to health and nutrition has raised growing concerns about the controversial effects of artificial colorants widely used in the food industry (Sebastian et al. 2023; Xavier and Mercadante 2019).

The established link between dietary habits, health, and longevity has driven a profound shift in consumer behavior, strengthening the demand for safer, more natural, and functional food ingredients. Scientific evidence associating artificial colorants with allergenic reactions, hyperactivity in children, and potential mutagenic or carcinogenic effects has further intensified this movement. As a result, consumers, researchers, and the food sector have redirected their efforts toward identifying natural alternatives capable of providing both technological and health‐related benefits (Akintunde et al. 2021; Ramesh and Muthuraman 2018; Urnau et al. 2018; Weiss et al. 2023).

In this scenario, carotenoids, natural pigments found in plants, algae, and microorganisms, have attracted increasing scientific and industrial interest. Beyond imparting characteristic colors to foods, these compounds exhibit important functional properties, including antioxidant activity and provitamin A activity, which make them promising ingredients for applications in the food, pharmaceutical, nutraceutical, and cosmetic industries (Nabi et al. 2023; Singh et al. 2023; Urnau et al. 2019).

Despite their advantages, carotenoids are highly susceptible to environmental stressors such as light, oxygen, and elevated temperatures, which may cause degradation through isomerization and oxidation. In addition, their lipophilic nature limits their direct incorporation into aqueous food systems, representing a significant technological barrier to broader application (Campo et al., 2019; Lu et al. 2020).

To mitigate these limitations, protective strategies are required to preserve the stability and functionality of carotenoids. Among the available technologies, spray‐drying microencapsulation has emerged as an efficient and versatile approach (Eun et al. 2020). In this process, a solution containing the bioactive compound and a wall material is atomized to form solid microstructures that act as physical and chemical barriers. This technique not only improves stability against environmental degradation but can also enhance dispersibility and functionality in different food systems (Jafari et al. 2023).

Most research on spray drying encapsulation has focused on carotenoids of plant origin (Tupuna et al. 2018; Corrêa‐Filho et al. 2019; Elik et al. 2021; Lima et al. 2021; Šeregelj et al. 2021; Pinho et al. 2022). Although studies have explored the production of carotenoid pigments from microorganisms using agro‐industrial residues as production substrates (Colet et al. 2015, 2019; Griep et al. 2023; Menegazzi et al. 2020), studies addressing the subsequent stabilization of these pigments through microencapsulation and their effective application in food matrices remain limited.

Furthermore, the literature reveals important gaps that limit the consolidation of microbial carotenoids as competitive alternatives to synthetic and plant‐derived pigments. There is a clear lack of integrated studies that simultaneously evaluate (i) sustainable microbial production using agro‐industrial by‐products, and (ii) stability after spray‐drying encapsulation. Existing investigations are generally fragmented, focusing either on pigment biosynthesis or on basic physicochemical characterization, without advancing toward comprehensive stability assessment and functional application.

In response to these gaps, the present study proposes a novel and integrated approach by combining the biotechnological production of carotenoids by *Sporidiobolus salmonicolor* CBS 2636 using agro‐industrial residues, under conditions previously established in earlier publications (Colet et al. 2017; Valduga et al. 2009), with their subsequent microencapsulation via spray drying (Griep et al. 2023) and evaluation for potential application in a food matrix.

This strategy aligns with the principles of sustainability and circular economy, as it simultaneously replaces synthetic colorants with natural alternatives and valorizes agro‐industrial residues, reducing environmental impacts and production costs (Amore et al. 2019; Colet et al. 2017). Thus, this work advances the current state of knowledge by establishing an underexplored route that connects sustainable pigment production, stabilization technology, and the technological characterization of the resulting microcapsules as potential food ingredients.

The selection of GA, inulin IN, and ST as wall materials was based on their complementary physicochemical and functional properties, which are particularly suitable for the encapsulation of lipophilic compounds such as carotenoids. GA is widely recognized for its excellent emulsifying capacity, high water solubility, and film‐forming ability, which favor emulsion stabilization and improve encapsulation efficiency during spray drying (Corrêa‐Filho, Moldão‐Martins, et al. 2019; Gharsallaoui et al. 2007; Labuschagne 2018). IN, a low‐molecular‐weight, presents low intrinsic viscosity, prebiotic functionality, and reduced hygroscopicity, characteristics that can contribute to improved powder stability and technological performance in food systems (Corrêa‐Filho, Moldão‐Martins, et al. 2019). ST, in turn, provides structural reinforcement to the encapsulating matrix due to its film‐forming and gel‐forming properties, contributing to particle integrity and reduction of water mobility within the matrix (Hoyos‐Leyva et al. 2018; Jafari et al. 2008).

Although gum arabic‐based systems have been widely applied for carotenoid encapsulation by spray drying, most studies rely on single or binary wall materials. In contrast, the present study proposes a statistically optimized ternary matrix (GA–IN–ST) to combine emulsification capacity (GA), reduced hygroscopicity and functional attributes (IN), and structural reinforcement (ST), aiming to improve encapsulation performance and thermal protection through synergistic effects.

Given the well‐known susceptibility of carotenoids to degradation induced by oxygen, light, heat, and acidic conditions, this study aimed to encapsulate carotenoid‐rich extracts produced by *S. salmonicolor* via spray‐drying using the GA–IN–ST system. The process was systematically optimized through a 2^3^ central composite rotational design (CCRD), enabling the identification of an optimal compositional range based on predictive modeling. The resulting microcapsules were characterized regarding their structural, physicochemical, morphological, and thermal properties, providing a technological basis for their application as stable natural food colorants.

## 2 Materials and Methods

### 2.1 Production and Recovery of Carotenoids

Carotenoid production was carried out in a Biostat B 5‐L fermentor (Braun Biotech International) operated as a semicontinuous system using the yeast *S. salmonicolor* CBS 2636 (Centraalbureau voor Schimmelcultures, Utrecht, The Netherlands), according to Colet et al. (2017). The initial culture medium (1 L, 10% (v/v) inoculum) contained 80 g/L crude glycerol, 80 g/L corn steep liquor, and 2 g/L rice parboiling water. Operating conditions were pH 4.0, 25°C, 180 rpm, 1.5 vvm aeration rate, and a bioproduction time of 96 h. Every 96 h, 50 % of the culture volume was removed and replaced with fresh sterilized medium, totaling 288 h of operation with three renewal cycles.

Carotenoid extraction and recovery followed Valduga et al. (2009), with modifications. The fermentation broth was centrifuged (4534 × g, 4°C, 10 min) using a centrifuge (MPW‐351R; MPW Med. Instruments, Poland) and washed with distilled water and petroleum ether (Dinâmica) to remove glycerol. Cell disruption was carried out by maceration in liquid nitrogen. After the addition of dimethyl sulfoxide (Nuclear) (2:1, v/w), the mixture was heated at 55°C for 30 min in an ultrasonic bath (USC‐1800A, Unique UltraSonic Cleaner, Brazil). Extraction was performed with acetone (Vetec): methanol (Quimex) (7:3, v/v) and successive centrifugations. Supernatants were collected repeatedly until the cells became colorless. The extract was stored at 3°C in an air flow system refrigerator DC47 (Electrolux, Brazil) or lyophilized using a Modulyo freeze dryer (Edwards, UK) coupled to a vacuum pump RV8 (Edwards, UK) for 48 h prior to further analysis.

#### 2.1.1 Total Carotenoids

Total carotenoid concentration was estimated from the absorbance measured at 448 nm, according to Equation 1 (Davies 1976). The extinction coefficient used was that of β‐carotene in methanol, *E* ^1%^_1 cm_ = 2550 (Schmalzer et al. 2008). Results were expressed as total carotenoids (µg/L).

(1) $$\mathrm{Ct}=\frac{Ey}{E_{1cm}^{1\%}\;x\;100}x\;10^{6}$$

where: *C_t_* is the total carotenoids (µg/L); *y* is the solution volume (mL); *E* is the absorbance at 448 nm; *E* ^1%^_1 cm_ is the extinction coefficient for β‐carotene (*E* ^1%^_1 cm_ = 2550).

### 2.2 Encapsulation of Carotenoids by Spray Drying

The organic solvents were completely removed under reduced pressure prior to the preparation of the emulsions. The concentrated carotenoid extract, free of residual acetone and methanol, was subsequently dispersed into the aqueous phase containing the previously hydrated wall materials.

Initially, the composition of the encapsulation matrix was defined through preliminary trials with different wall materials and proportions: MD—Maltodextrin (Ingredientes Online); GA—Gum arabic (Vetec); WPI60%—Whey protein isolate (at 60% concentration) (SOORO Ingredientes); WPI80%—Whey protein isolate (at 80% concentration) (SOORO Ingredientes); XG—Xanthan gum (Dinâmica); TSP—Textured soy protein (Duplogel MF Marsul); ST—Starch P.A. (Synth); MS—Modified starch (Ingredion); TR—Trehalose (Exato); IN—Inulin (Ingredientes Online); PC—Pure casein (Ingredientes Online); SC—Sodium caseinate (Sigma‐Aldrich); FOS—Fructooligosaccharide (Ingredientes Online).

The experiments (Table 1) were conducted by dissolving wall materials in phosphate buffer pH 7.0 and homogenizing with a mechanical stirrer RW 20 digital (IKA, Germany) at 300 rpm for 5 min, followed by the addition of polysorbate 80 (Tween 80; Synth) (2%–4%, w/v) and carotenoid extract (5%–50%, v/v). The emulsion was subjected to ultrasonic treatment using an ultrasonic bath (Unique UltraSonic Cleaner, USC‐1800A, Brazil) operating at a frequency of 40 kHz and nominal power of 132 W for 30 min. The temperature was maintained at 50°C during the process to ensure adequate hydration of the wall materials. The final solution was protected from light, and its viscosity was measured prior to spray drying.

**TABLE 1:** Encapsulation efficiency (EE, %), product yield (EY, %), and solution viscosity (cP) of microcapsules obtained with different wall material formulations.

Runs	Wall material	EE (%)*	EY (%)*	Viscosity (cP)*
1	MD:GA:WPI_60%_	6.80^n^ ± 1.29	52.28^n^ ± 0.69	2.08^bcdef^ ± 0.02
2	MD:GA:WPI_80%_	0.00^n^ ± 0.00	63.28^ij^ ± 0.63	2.16^bcdef^ ± 0.02
3	MD:WPI_60%_	0.00° ± 0.00	46.04° ± 0.46	1.68^bcdefg^ ± 0.02
4	GA:WPI_60%_	9.72° ± 1.06	52.28^n^ ± 0.52	2.54^b^ ± 0.03
5	MD:XG	33.88^jkl^ ± 0.28	33.43^q^ ± 0.33	109.89^a^ ± 1.09**
6	MD:GA:TSP	37.61^ijk^ ± 2.24	66.42^gh^ ± 0.66	2.14^bcdef^ ± 0.02
7	GA:TSP	27.02 ^lm^ ± 3.12	52.78^n^ ± 0.52	2.36^bcde^ ± 0.02
8	MD:TSP	40.38^hij^ ± 3.76	57.53^l^ ± 0.57	1.74^bcdefg^ ± 0.02
9	MD:GA:ST	67.58^ab^ ± 2.39	79.43^bc^ ± 0.79	2.00^bcdefg^ ± 0.02
10	MD:GA:MS_2_	51.90^defg^ ± 1.65	67.96^fg^ ± 0.67	2.11^bcdef^ ± 0.02
11	MD:GA:ST+T	0.00^n^ ± 0.00	82.14^ab^ ± 0.81	2.42^bc^ ± 0.02
12	MD:ST	69.37^ab^ ± 0.48	30.58^rs^ ± 0.30	1.70^bcdefg^ ± 0.02
13	GA:ST	61.28^bcd^ ± 4.67	76.42^de^ ± 0.76	2.32^bcde^ ± 0.02
14	MD:GA:ST:TSP	47.64^efghi^ ± 5.56	64.38^hi^ ± 0.64	1.91^bcdefg^ ± 0.02
15	MD:GA:TR	46.91^fghi^ ± 3.38	74.56^e^ ± 0.74	1.85^bcdefg^ ± 0.02
16	MD:TR	0.00^n^ ± 0.00	12.36^u^ ± 0.12	1.54^cdefg^ ± 0.02
17	GA:TR	39.11^ijk^ ± 4.86	55.84 ^lm^ ± 0.55	2.04^bcdef^ ± 0.02
18	MD:GA:TR:ST	54.59^cdef^ ± 1.30	27.86^s^ ± 0.28	1.64^bcdefg^ ± 0.02
19	MD:GA:TR:TSP	22.43^m^ ± 0.51	30.54^rs^ ± 0.30	1.84^bcdefg^ ± 0.02
20	GA:TR:ST	73.43^a^ ± 2.76	84.16^a^ ± 0.83	1.56^cdefg^ ± 0.02
21	MD:GA:IN	62.80^bc^ ± 2.07	64.54^hi^ ± 0.64	1.44^efg^ ± 0.01
22	MD:IN	29.29^klm^ ± 2.23	23.72^t^ ± 0.23	1.11 ^g^ ± 0.01
23	GA:IN	68.26^ab^ ± 0.79	61.08^jk^ ± 0.60	1.66^bcdefg^ ± 0.02
24	MD:GA:IN:ST	69.36^ab^ ± 1.45	60.36^k^ ± 0.60	1.93^bcdefg^ ± 0.02
25	MD:GA:IN:TSP	42.57^ghij^ ± 1.31	48.32° ± 0.48	1.91^bcdefg^ ± 0.02
26	GA:IN:ST	76.15^a^ ± 0.40	74.74^e^ ± 0.74	1.54^cdefg^ ± 0.02
27	MD:GA:PC	50.35^efgh^ ± 2.30	47.23° ± 0.47	1.48^defg^ ± 0.01
28	MD:GA:SC	6.59^n^ ± 2.71	78.12 ^cd^ ± 0.77	1.92^bcdefg^ ± 0.02
29	MD:PC	2.29^n^ ± 0.29	30.74^qr^ ± 0.30	1.28^fg^ ± 0.01
30	GA:PC	0.70^n^ ± 0.42	39.25^p^ ± 0.39	1.88^bcdefg^ ± 0.02
31	GA:ST:PC	2.48^n^ ± 0.48	58.50^kl^ ± 0.58	2.38^bcd^ ± 0.02
32	GA:ST:PC:IN	45.73^fghi^ ± 3.73	53.65^mn^ ± 0.53	1.89^bcdefg^ ± 0.02
33	MD:F0S:TR:WPI_60%_:ST	57.35^cde^ ± 0.66	60.34^k^ ± 0.60	1.56^cdefg^ ± 0.02

*Means ± standard error (n = 3) followed by the same lowercase on the collum do not differ significantly (p < 0.05) by one‐way analysis (Tukey test). Drying conditions: Atomization pressure of 0.08 – 0.12 bar, average feed flow rate of 5.83 mL/min, inlet air temperature of 120°C. Viscosity—Spindle ULA at 100 rpm, 25°C, except for **Run 5 (MD: XG), measured at 5 rpm.

Spray drying conditions were previously defined by the group (Griep et al. 2023): spray dryer SD‐05 (LabPlant, UK), 0.5 mm nozzle, inlet air temperature of 120°C, atomization pressure 0.08–0.12 bar, and an average feed rate of 5.83 mL/min. The encapsulated powder was stored in amber glass bottles wrapped in aluminum foil and kept at 22 ± 2°C in a desiccator until analysis (EE% and EY%).

Based on EE% results, a 2^3^ CCRD (Table 3) was performed to optimize the encapsulation matrix composition. The proportions of 20% (v/v) carotenoid extract and 78% (v/v) phosphate buffer (pH 7.0) refer to the liquid phase composition during emulsion preparation. The carotenoid extract was previously concentrated and solvent‐free prior to incorporation into the aqueous phase. Tween 80 was added at 2% (w/v) as an emulsifier. The wall materials (gum Arabic, inulin, and starch) were dissolved in the aqueous phase at concentrations defined by the experimental design (5–30 g/L each), and these concentrations represent the main contribution to the total solid content of the feed solution. Therefore, the total solid content of the final feed consisted of the sum of the wall materials (variable according to the design), the solid fraction of the carotenoid extract (core material), and 2% (w/v) Tween 80. Dependent variables (responses) included solution viscosity, EE, EY, water activity, and the moisture content of the microcapsules.

From the optimized condition, the morphological, structural, and functional characteristics of the wall materials and/or the microcapsules were also evaluated by scanning electron microscopy (SEM), X‐ray diffraction (XRD), Fourier‐transform infrared spectroscopy (FTIR), thermogravimetric analysis (TGA), and differential scanning calorimetry (DSC), as well as conductivity, resistivity, thermal diffusivity, and bulk density.

### 2.3 Characterization of Wall Materials and Microcapsules

#### 2.3.1 Encapsulation Yield

EY was determined from the total solid mass before microencapsulation (MSA) and the solid mass recovered after encapsulation (MSD), and was expressed as a percentage (%EY).

#### 2.3.2 Encapsulation Efficiency

Encapsulation efficiency was determined by quantifying surface and total carotenoids, as described by Griep et al. (2023). For surface carotenoids, 0.2 g of the sample was mixed with an ethanol (95% P.A., Química Moderna)/methanol (Quimex) solution (1:1, v/v) in a vortex mixer K40‐10208 (Kasvi, Brazil) for 10 s at 300 rpm, followed by centrifugation using a D‐78532 centrifuge (Hettich, Germany) at 10732 × g and 15°C for 20 min, and subsequent filtration through a 0.45 µm membrane (Millipore) prior to absorbance measurement using a spectrophotometer (UV‐1600 E; Pró‐Análise, Brazil) at 450 nm. Total carotenoids were quantified by complete disruption of the microcapsules with 2 mL of a methanol (Quimex), glacial acetic acid (99.8%, Neon), and water mixture (50:8:42, v/v/v), followed by vortex agitation (3000 rpm for 1 min), ultrasonic bath treatment (100 W for 40 min), centrifugation (10732 × g at 15°C for 20 min), filtration (Millipore, 0.45 µm), and spectrophotometric measurement at 450 nm. Results were expressed as encapsulation efficiency (%*EE*), using Equations 2 and 3.

(2) $$\mathrm{CS}\;\left(\%\right)=\frac{\mathrm{ABS}\;\mathrm{Surface}\;\mathrm{carotenoids}}{\mathrm{ABS}\;\mathrm{Total}\;\mathrm{carotenoids}}x\;100$$

(3) $$\mathrm{EE}\;\;\left(\%\right)=100-\mathrm{CS}\;\left(\%\right)$$

where: *CS* (%) is the percentage of carotenoids on the capsule surface, and *EE* (%) is the encapsulated carotenoid percentage.

#### 2.3.3 Viscosity

Viscosity was measured prior to spray drying using a rotational viscometer (Programmable DV‐III+ rheometer; Brookfield, USA) equipped with a ULA spindle, at 100 rpm and 25°C. A volume of 15 mL of sample was used, and readings were recorded in centipoise (cP).

#### 2.3.4 Moisture

Moisture content was determined gravimetrically by drying the samples in an air circulation oven at 105°C, according to method n°. 950.46 (AOAC 2000).

#### 2.3.5 Water Activity

Water activity (a*_w_*) of the microcapsules was determined using a water activity meter (Novasina AG, Switzerland) after sample stabilization at 27°C.

#### 2.3.6 Color Parameters (L*, a*, b*, Chroma C*)

The color parameters of free extracts and microcapsules were measured using a digital colorimeter (Chroma Meter CR‐400; Konica Minolta, Japan) in the CIELAB system: L* = lightness (0 = black to 100 = white), a* = chromaticity (−80 = green to +100 = red), b* = chromaticity (−50 = blue to +70 = yellow), and Chroma C* = color saturation.

#### 2.3.7 Scanning Electron Microscopy

The morphology of microcapsules was analyzed using SEM (EVO LS25; Zeiss, Germany). Samples were coated with gold and examined at an accelerating voltage of 10 kV. Micrographs were obtained with scale bars of 10 and 5 µm. The mean particle diameter was determined using Size Meter software (v.1.1) based on 100 particles.

#### 2.3.8 X‐Ray Diffraction

XRD patterns of wall materials and microcapsules were obtained using an X‐ray diffractometer (Miniflex II; Rigaku, Japan) with a copper tube (Cu Kα, λ = 1.54 Å) for crystallinity peak analysis.

#### 2.3.9 Fourier‐Transform Infrared Spectroscopy

FTIR spectra of wall materials, free carotenoid extract, and microcapsules were recorded using an FTIR spectrometer (Cary 630; Agilent Technologies, USA) equipped with a ZnSe ATR crystal. Spectra were acquired in the range of 4000–650 cm^−1^, at a resolution of 4 cm^−1^ with eight scans, using Happ‐Genzel apodization. The main absorption bands were identified, and the corresponding peak positions were recorded (cm^−1^).

#### 2.3.10 Thermogravimetric Analysis

TGA was performed using a TGA instrument (Q500; TA Instruments, USA). Samples were placed in aluminum pans and heated under nitrogen flow (50 mL/min), over a temperature range of 25°C to 500°C, at a heating rate of 10°C/min. All TGA analyses were conducted under nitrogen atmosphere to avoid oxidative degradation.

#### 2.3.11 Differential Scanning Calorimetry

Thermal properties were evaluated using a DSC instrument (DSC‐60; Shimadzu, Japan). Samples (5 mg) were sealed in aluminum pans and analyzed under a nitrogen flow (150 mL/min) with heating from 30 to 300°C at a rate of 10°C/min. All DSC analyses were also performed under nitrogen atmosphere to ensure inert thermal conditions.

#### 2.3.12 Thermal Conductivity, Resistivity, and Diffusivity

Measurements were performed at 22°C ± 2°C using a KD2 Pro probe (Decagon Devices, USA), with a diameter of 1.28 mm x a length of 60 mm.

#### 2.3.13 Bulk Density

The bulk density of the microcapsules was determined according to previous study.

### 2.4 Statistical Analysis

All results (*n* = 3) were analyzed using experimental design methodology and ANOVA at a 95 % confidence level (*p*‐values ≤ 0.05) to determine significant differences among sample means, using Statistica software, version 5.0 (StatSoft, Inc., USA).

## 3 Results and discussion

### 3.1 Optimization of Microencapsulation of Carotenoids

The GA:IN:ST (Run 26) and GA:TR:ST (Run 20) treatments showed the highest (*p* < 0.05) EE, of 76.15% and 73.43%, respectively (Table 1). Other encapsulating matrices also demonstrated potential for carotenoid encapsulation, including MD:ST (Run 12), MD:GA:IN:ST (Run 24), GA:IN (Run 23), and MD:GA:ST (Run 9), with EE values ranging from 67.58% to 69.36%, respectively. Maltodextin is known for its ability to form stable dry particles, although it has poor emulsifying properties (Gabriela et al. 2020). However, when combined with gum arabic, inulin, and corn starch, it showed promising results. Inulin has emulsifying and gelling properties that can improve the retention of encapsulated substances. In addition, trehalose is recognized as a bioprotective agent against various environmental factors. When combined with gum arabic and corn starch, which have strong emulsifying and stabilizing capacities, trehalose contributes to the formation of stable emulsions with low viscosity (Corrêa‐Filho et al. 2019; Eun et al. 2020; Gonçalves et al. 2017).

The surfactant Tween 80 had a significant effect on EE. When a concentration of 2% (w/v) Tween 80 was used (Run 9), EE reached 67.58% and the encapsulation yield reached 79.43% (Table 1), indicating a positive effect of the surfactant in stabilizing micelles and incorporating carotenoids.

Wall material properties, such as solubility, viscosity, and film‐forming ability, can affect encapsulation yield. Poorly soluble wall materials may not disperse adequately in the feed solution, leading to reduced yields (Correâ‐Filho et al. 2019; Medina‐Torres et al. 2013; Shao et al. 2018), as observed in the following treatments (Table 1) MD:TR (Run 16), MD:IN (Run 22), MD:GA:TR:ST (Run 18), and MD:ST (Run 12).

Runs with higher viscosity (Table 1), such as Run 4 (GA:WPI 60%—viscosity of 2.54 cP, EE of 9.72%), Run 11 (MD:GA:ST+T—viscosity of 2.42 cP, EE of 0.00%), and Run 31 (GA:ST:PC—viscosity of 2.38 cP, EE of 2.48%), exhibited the lowest EE. These results suggest that increased viscosity, promoted by materials such as proteins (WPI), GA, and Tween 80, may hinder micelle stabilization and carotenoid incorporation, resulting in lower EE. Moreover, high viscosity leads to larger, more elongated droplets, reducing core circulation and delaying the formation of the semipermeable membrane, thus producing larger, less efficient particles (Gharsallaoui et al. 2007; Jafari et al. 2008; Labuschagne 2018; Santos et al. 2021). It is important to note that Run 11, despite presenting higher viscosity than Run 31, resulted in EE = 0%. This result indicates that viscosity alone does not determine encapsulation efficiency. In this condition, the formulation likely exceeded the optimal viscosity range, which may have compromised emulsion stability and atomization during spray drying. Therefore, EE depends on the combined effects of viscosity, wall material proportion, and drying behavior.

Based on the results presented in Table 1, GA:IN:ST (Run 26) was selected for further experiments. Although the trehalose combination (Run 20) also performed well in terms of EE and EY, IN was chosen for its more suitable functional profile. Its prebiotic properties, emulsifying and gelling capacities, and lower hygroscopicity favor its use in food applications (Corrêa‐Filho et al. 2019).

Once the wall materials were defined, new runs (Table 2) were performed by varying the carotenoid extract concentration while keeping the drying temperature (120°C), Tween 80 concentration (2%, w/v), and GA:IN:ST ratio (1:1:1, w/w/w) constant. Runs with 5% and 20% (v/v) carotenoid extract yielded the highest (*p* < 0.05) EE values. Interestingly, despite a fourfold increase in extract concentration in Run 2 compared to Run 1, no significant EE improvement was observed. Regarding yields, Run 2 achieved a higher EY (84.69%) than the others runs (*p* < 0.05), suggesting that 20% (v/v) extract concentration did not compromise efficiency and enhanced EY.

**TABLE 2:** Emulsion viscosity (cP), encapsulation efficiency (EE, %), and product yield (EY, %) of microcapsules produced with GA:IN:ST (1:1:1, w/w/w) as wall material and different carotenoid extract concentrations (%, v/v).

Run	Extract (% v/v)	Viscosity (cP)*	EE (%)*	EY (%)*
1	5	1.54^d^± 0.02	76.85^a^ ± 0.40	74.74^b^± 0.74
2	20	1.99^c^± 0.02	75.55^a^ ± 1.17	84.69^a^ ± 0.84
3	30	2.33^b^ ± 0.02	52.62^b^ ± 5.72	52.90^c^ ± 0.52
4	40	2.77^a^ ± 0.03	55.23^b^ ± 4.77	74.84^b^ ± 0.78

*Means ± standard error (n = 3) followed by the same lowercase on the collum do not differ significantly (p < 0.05) by one‐way analysis (Tukey test). Carotenoid extract concentration expressed as % (v/v) relative to feed solution. Drying conditions: inlet air temperature 120°C, atomization pressure 0.08–0.12 bar, feed flow rate 5.83 mL/min. Viscosity measured at 25°C, spindle ULA, 100 rpm.

In general, emulsion viscosity increased as extract concentration increased, due to the higher solute content in the feed solution. This relationship influences particle stability during encapsulation since higher viscosities may hinder droplet formation and stabilization.

In summary, a 20% (v/v) extract concentration (Table 2) is recommended to maximize both encapsulation efficiency and yield. This condition ensured a proportional relationship between extract concentration and encapsulated content, while emulsion viscosity (1.99 cP) remained suitable for pumping at an average feed flow of 5.83 mL/min and atomization with a 0.5 mm nozzle in the spray dryer.

Based on these results (Table 2), the wall materials (GA, IN, and ST), Tween 80 concentration (2%, w/v), and carotenoid extract concentration (20%, v/v) were established for the optimization stage of carotenoid microencapsulation.

**FIGURE 1:**
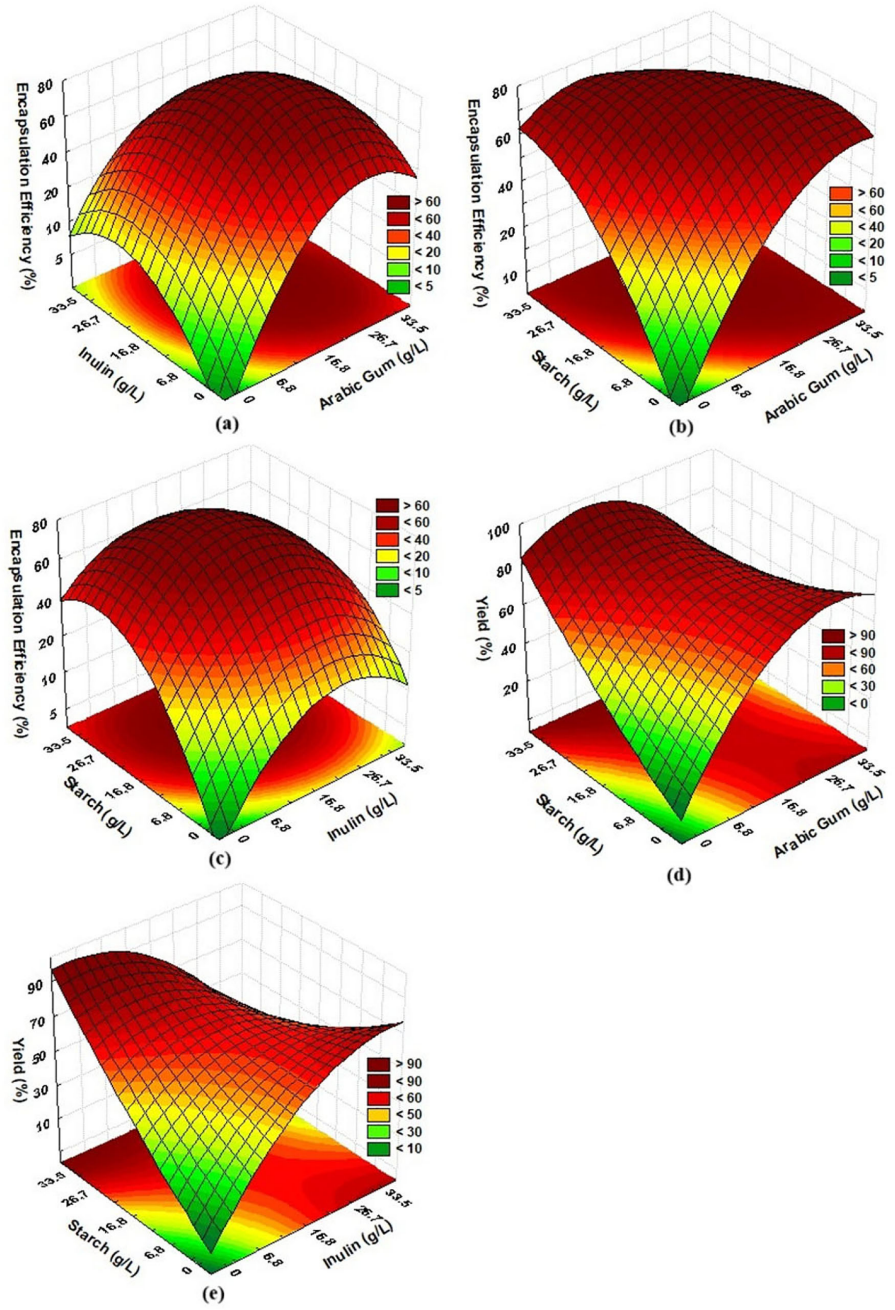
Response surface plots obtained from the central composite design. (a) The interaction between GA and inulin reveals a region where increasing GA enhances EE, while excessive IN reduces it due to matrix weakening. (b) For GA and starch, higher ST proportions tend to increase EE up to an intermediate range, after which viscosity‐induced diffusion limitations diminish the effect. (c) The combination of IN and ST shows that balanced ratios promote better structural integration of the wall materials, enhancing EE. (d) Yield (YE) as a function of GA and ST indicates that formulations richer in GA improve drying efficiency. (e) YE as a function of IN and ST demonstrates that excessive starch compromises yield due to poor film formation, while moderate IN levels stabilize the matrix, resulting in improved recovery.

Table 3 presents the matrix of the 2^3^ CCRD, containing the coded and real values of the independent variables (concentration of GA, IN, and ST) studied in the microencapsulation of carotenoids and the dependent variables (responses) in terms of emulsion viscosity, EE (predicted, deviation, and relative deviation), EY, a*_w_*, and encapsulate moisture content. The results (Table 3) were statistically analyzed (*p* < 0.05), and Equation 4 (Table 4) presents the second‐order coded model that describes the relationship between carotenoid EE and the studied variables (concentration of GA, IN, and ST) within the experimental range. The model was validated by ANOVA, with an R of 0.90 and an F‐calculated value that was 1.36 times higher than the tabulated value, which also allowed the construction of response surfaces (Figure 1a–c). The regression model was statistically significant (*p* < 0.05), with an adjusted R^2^ of 0.81, indicating adequate predictive capacity. Additionally, the lack‐of‐fit test was significant (*p* > 0.05), confirming that the model properly describes the experimental data within the studied range.

**TABLE 3:** Central composite rotational design (2^3^ CCRD) matrix (coded and actual values) and responses for microcapsules produced with varying concentrations of GA, IN, and ST (g/L), including encapsulation efficiency (EE, %), predicted EE, deviation, relative deviation (%), product yield (EY, %), viscosity (cP), water activity (a*_w_*), and moisture content (%).

Independent variables				Responses							
Runs	GA (g/L)	IN (g/L)	ST (g/L)	EE_experimental_ (%)	EE_predicted_ (%)	Deviation	Relative deviation (%)	EY (%)	Viscosity (cP)	a*_w_*	Moisture (%)
**1**	−1 (6.8)	−1 (6.8)	−1 (6.8)	22.70	−2.53	25.23	111.14	17.34	1.31	0.219	8.15
**2**	1 (26.7)	−1 (6.8)	−1 (6.8)	64.10	61.11	2.99	4.66	39.34	2.74	0.196	8.06
**3**	−1 (6.8)	1 (26.75)	−1 (6.8)	19.65	18.95	0.70	3.57	54.00	2.08	0.190	7.57
**4**	1 (26.7)	1 (26.7)	−1 (6.8)	68.53	62.19	6.34	9.25	80.85	2.99	0.147	5.54
**5**	−1 (6.8)	−1 (6.8)	1 (26.7)	58.09	55.65	2.44	4.20	85.68	2.43	0.127	5.15
**6**	1 (26.7)	−1 (6.8)	1 (26.7)	69.20	61.13	8.07	11.66	91.38	3.41	0.181	7.33
**7**	−1 (6.8)	1 (26.7)	1 (26.7)	65.71	59.93	5.78	8.79	88.89	2.47	0.104	5.18
**8**	1 (26.7)	1 (26.7)	1 (26.7)	28.55	45.01	−16.46	−57.64	50.39	2.75	0.135	5.26
**9**	−1.68 (0)	0 (16.7)	0 (16.7)	4.79	20.92	−16.13	−336.61	40.03	2.25	0.210	8.38
**10**	1.68 (33.5)	0 (16.7)	0 (16.7)	65.54	61.85	3.69	5.63	71.28	2.95	0.162	5.87
**11**	0 (16.7)	−1.68 (0)	0 (16.7)	29.51	48.33	−18.82	−63.78	69.37	2.48	0.223	7.94
**12**	0 (16.7)	1.68 (33.5)	0 (16.7)	59.26	52.84	6.43	10.84	55.19	2.80	0.168	5.93
**13**	0 (16.7)	0 (16.7)	−1.68 (0)	14.88	31.67	−16.79	−112.86	93.77	2.37	0.221	8.12
**14**	0 (16.7)	0 (16.7)	1.68 (33.5)	70.46	66.11	4.35	6.17	89.58	3.11	0.102	5.37
**15**	0 (16.7)	0 (16.7)	0 (16.7)	76.77	75.14	1.63	2.12	79.52	2.71	0.125	5.61
**16**	0 (16.7)	0 (16.7)	0 (16.7)	75.90	75.14	0.76	1.00	81.26	2.54	0.115	5.24
**17**	0 (16.7)	0 (16.7)	0 (16.7)	75.30	75.14	0.16	0.21	83.85	2.66	0.123	5.67
**18**	0 (16.7)	0 (16.7)	0 (16.7)	74.84	75.14	−0.30	−0.40	80.36	2.79	0.131	5.46
**19**	0 (16.7)	0 (16.7)	0 (16.7)	75.12	74.14	−0.02	−0.03	91.93	2.69	0.138	5.51

Fixed formulation components: carotenoid extract 20 % (v/v), phosphate buffer (pH 7.0) 80 % (v/v), Tween 80 (2 %, w/v). Units: EE, EY, deviation and relative deviation expressed in %, viscosity measured in cP, water activity as aw, moisture expressed in %.

**TABLE 4:** Second‐order coded models, correlation coefficients (R), and F‐values (F_cal_ > F_tab_) describing encapsulation efficiency (EE, %), encapsulation yield (EY, %), solution viscosity (cP), water activity (a*_w_*), and moisture content (%) as a function of gum arabic (GA), inulin (IN), and starch (ST) concentrations (g/L).

Second‐order coded models	R	R^2^	R^2^adj	F_cal_ > F_tab_	Df (Reg/Res)	Lack‐of‐fit (Df)	*p*‐value (model)	Eq.
$\mathrm{EE}\;(\%)=75.14+12.18\;X_{1}\;-\;11{.96\;X}_{1}^{2}+1.34\;X_{2}\;-\;8.7{.\;X}_{2}^{2}+10.25\;X_{3}-9.30\;X_{3}^{2}\;-\;5.10\;X_{1}.X_{2}\;-\;14.54\;X_{1}.X_{3}-4.30\;X_{2}.\;X_{3}$	0.90	0.81	0.62	1.36	9/9	5/4	0.002	(4)
$\mathrm{YE}\;(\%)=83.61+5.02\;X_{1}\;-\;11{.01\;X}_{1}^{2}\;-\;8{.66\;X}_{2}^{2}+8.63\;X_{3}-10.20\;X_{1}.X_{3}-14.49\;X_{2}.\;X_{3}$	0.87	0.76	0.64	1.67	6/12	8/4	0.004	(5)
$\mathrm{Visc}\;(\mathrm{cP})=2.68+0.350\;X_{1}+0.069X_{2}+0.233\;X_{3}-0.152\;X_{1}.X_{2}-0.135\;X_{1}.X_{3}-0.205\;X_{2}.\;X_{3}$	0.94	0.88	0.83	5.85	6/12	8/4	<0.001	(6)
$a_{w}=0.127+0.016X_{1}^{2}\;-\;0.018X_{2}+0.02X_{2}^{2}\;-\;0.03X_{3\;}+0.008X_{3}^{2}+0.019X_{1}.X_{3}$	0.94	0.88	0.83	5.00	6/12	8/4	<0.001	(7)
$M\;(\%)=5.52\;-\;0.30X_{1}+0.47X_{1}^{2}\;-\;0.62X_{2}+0.40X_{2}^{2}\;-\;0.81\;X_{3\;}+0.34X_{3\;}^{2\;}-0.50X_{1}.X_{2}+0.55X_{1}.X_{3}$	0.95	0.902	0.821	3.44	8/10	6/4	<0.001	(8)

X1 = GA (g/L); X2 = IN (g/L); X3 = ST (g/L). EE, EY, moisture expressed in %, viscosity in cP, water activity as aw. Statistical parameters: R—correlation coefficient; Fcal—calculated F‐value; Ftab—tabulated F‐value; Degrees of freedom (df) (Regression / Residual).

The response surfaces allow the identification of a region of maximum EE, located between 10 and 26 g/L of GA, IN, and ST, thus demonstrating the optimization of the carotenoid encapsulation process. In this concentration range, low relative deviations (< 2%) were observed, showing that the second‐order coded model adequately fits the experimental data.

Thus, when using GA:IN:ST 1:1:1 w/w/w (16.7 g/L) as wall materials, there is an increase of approximately 17% in encapsulation efficiency when compared with the previous study by Griep et al. (2023), which used GA:MD 1:1 w/w (25 g/L). This increase in efficiency may be attributed to the specific properties of these materials, such as the ability of gum arabic to form encapsulating films and entrap carotenoids, in addition to the synergistic contributions of inulin and corn starch in the formation of the polymeric matrix.

In another study that investigated the encapsulation of annatto‐derived norbixin, the most efficient condition was MD:GA (0:100 w/w) using 30 g of norbixin, resulting in an encapsulation efficiency of 75%. In that context, the positive influence of GA as a wall material was highlighted, emphasizing its emulsifying properties and film‐forming capacity, both of which are essential for compound retention within microcapsules (Tupuna et al. 2018). Another study by Álvarez‐Henao et al. (2018) reported the encapsulation of lutein using MD:GA:ST (33.3:33.3:33.3 w/w/w), achieving an encapsulation efficiency of 65.72%.

The highest EY values (Table 3) were 93.77% (Run 13), 91.93% (Run 19), and 91.80% (Run 6). From an industrial perspective, EY values between 70% and 90% are considered economically viable and satisfactory for many applications (Tontul and Topuz 2017; Tupuna et al. 2018). The second‐order coded model (Equation 5, Table 4) describes the relationship between the EY of carotenoid capsules and the independent variables (GA, IN, and ST concentrations) within the studied range. The model was validated by ANOVA, which also allowed the construction of response surfaces (Figure 1). Figure 1d and 1e show the response surfaces, indicating that yield is higher when the GA concentration is maintained at low levels (approximately 6.8–16.8 g/L), while the ST concentration remains high (above 30 g/L) and the IN concentration remains low (up to approximately 11 g/L), in combination with high ST concentrations (above 30 g/L). This demonstrates a region of yield maximization at specific combinations of the independent variables within the studied range.

In a previous study, the encapsulation of carotenoid, rich extracts produced by *S. salmonicolor* using GA and ST resulted in yields ranging from 41.94% to 56.11%. The highest value was obtained when using an inlet drying temperature of 130°C and a wall matrix composed of 37.6 g/L GA, 25 g/L MD, 2% (w/v) Tween 80, and 5% (v/v) concentrated carotenoid extract (Griep et al. 2023). In comparison with the results of the present study, a significant improvement in this parameter was observed.

One of the main causes of losses of encapsulated material is powder deposition on the walls of the drying chamber. In addition, high emulsion viscosity hinders the proper formation of fine droplets. Another factor contributing to low yields is the outlet angle of the particles from the nozzle, which may cause collisions with the inner walls of the atomization cylinder. These collisions lead to material loss and, consequently, to a reduction in mass yield efficiency (Correâ‐Filho et al. 2019; Ramakrishnan et al. 2018).

**FIGURE 2:**
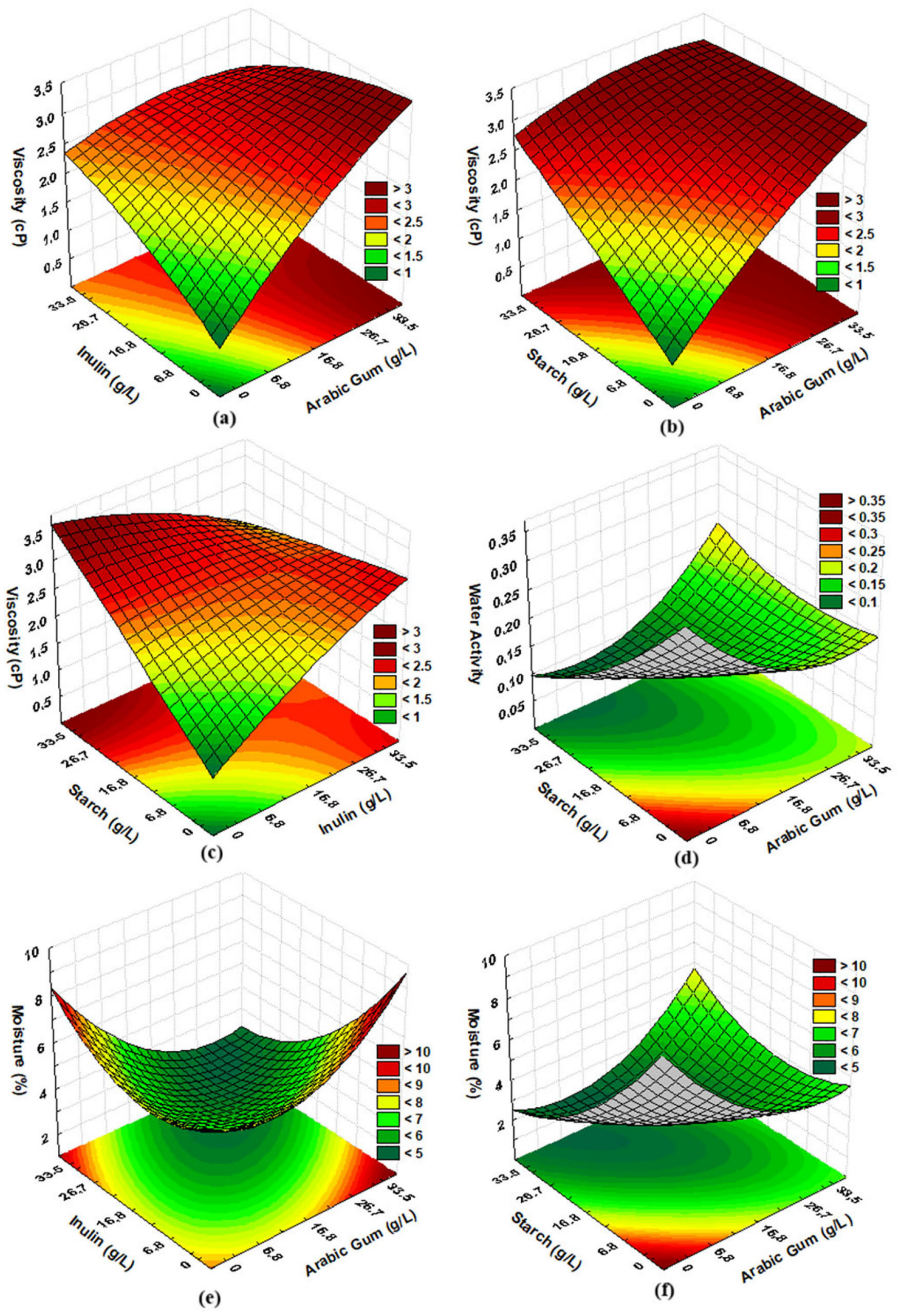
Response surface plots describing key physicochemical parameters of the encapsulation formulations. (a) Solution viscosity as a function of GA and IN shows that GA predominantly increases viscosity, while IN contributes moderately, influencing droplet formation during drying. (b) GA combined with ST results in a strong viscosity rise at higher starch levels, reflecting granule swelling and increased molecular entanglement. (c) IN and ST together demonstrate synergistic effects, generating intermediate viscosities favorable for atomization. (d) Water activity (aw) as a function of GA and ST decreases with higher GA levels, indicating improved water binding and reduced free water mobility. (e) Moisture content influenced by GA and IN reveals that inulin‐rich formulations retain slightly more water due to its hygroscopic nature. (f) GA and ST interactions show that increased starch promotes higher residual moisture, consistent with slower water diffusion during drying.

Emulsion viscosity directly impacts droplet formation and size, encapsulation efficiency, and the quality of the resulting microcapsules. Ideally, moderate viscosity is desired, as it facilitates the formation of spherical and homogeneous droplets during atomization while avoiding issues such as nozzle clogging (Santos et al. 2021). The emulsion viscosity ranged from 1.31 to 3.41 cP (Table 3). Equation 6 (Table 4) presents the first‐order coded model describing the relationship between emulsion viscosity and the independent variables within the studied range. The model was validated by ANOVA and also allowed the construction of response surfaces (Figure 2a–c).

This suggests that higher amounts of wall materials contribute to the formation of a viscous matrix, as expected due to the thickening properties of these polysaccharides. An increase in viscosity may favor encapsulation efficiency when within an optimal range, as moderate viscosity contributes to emulsion stabilization and reduction of droplet coalescence prior to spray drying. However, excessively high viscosity may impair atomization efficiency and droplet formation, negatively affecting encapsulation performance.

However, very high concentrations of wall materials may excessively increase viscosity, delaying particle formation during drying, which implies greater exposure during atomization and higher losses of the compound of interest (Correâ‐Filho et al. 2019; Gharsallaoui et al. 2007; Mohammed et al. 2017). Accordingly, GA (Figure 2a), when used at higher concentrations, resulted in more viscous solutions. This relationship can be attributed to the ability of gums to form a viscoelastic matrix, increasing molecular cohesion and thus viscosity (Lima et al. 2021). Similar results were observed in previous studies, where high GA concentrations led to more viscous solutions (Premi and Sharma 2017).

IN, although known for its low intrinsic viscosity, slightly increased solution viscosity when included in the formulation. ST, a polysaccharide widely used as a wall material in encapsulation, also increased viscosity as its concentration increased, which can be explained by its gel‐forming ability at higher concentrations. During gelatinization, macromolecular entanglement and amylose solubilization occur, contributing to an increase in solution viscosity.

The improved encapsulation performance can be mechanistically explained by the complementary roles of the wall materials. GA contributes primarily to emulsion stabilization due to its surface‐active properties. IN reduces hygroscopicity and contributes to matrix densification, while starch enhances structural integrity through film‐forming and gel‐forming behavior. The synergistic combination of these polysaccharides likely promotes enhanced matrix cohesion and improved protection of the carotenoid core during rapid solvent evaporation in spray drying.

However, viscosity decreased when GA and IN were used at equal concentrations, suggesting a synergistic interaction between the two materials. A possible explanation lies in the intrinsic characteristics of the wall materials: GA has a high molecular weight and a branched structure, conferring a high water retention capacity and the formation of a three‐dimensional network. In contrast, IN has a low molecular weight and a linear structure, resulting in lower water retention and limited network formation. Therefore, the interaction between these two materials may balance their properties, resulting in an intermediate viscosity.

The relationship between viscosity and encapsulation efficiency is not strictly linear. While moderate viscosity enhances emulsion stability and reduces droplet coalescence, excessively high viscosity may impair atomization efficiency, leading to suboptimal droplet formation and reduced encapsulation performance.

The stability of encapsulated bioactive compounds is influenced by several parameters, such as a*_w_* and moisture content. In environments with low a*_w_* (up to 0.25), minimal lipid oxidation, little enzymatic activity, and limited browning reactions are expected. When a*_w_* is between 0.3 and 0.5, powder agglomeration tends to occur, whereas values above 0.6 favor the growth of molds, bacteria, and yeasts (Janiszewska‐Turak et al. 2017; Lim and Roos 2017).

The a*_w_* values ranged from 0.102 to 0.223 (Table 3), which were considerably lower than typical values reported in the literature. In contrast, the moisture content varied between 5.15% and 8.38%. Moisture levels play a crucial role in preserving the functional properties and overall quality of the final product. Previous studies suggest that when the moisture content falls below 7%, water diffusion through the food matrix is reduced, thereby minimizing its impact on physicochemical properties and restricting oxygen accessibility within the microcapsule structure (Liu et al. 2022; Zhang et al., 2019).

Equations 7 and 8 (Table 4) present the second‐order coded models for a*_w_* and moisture, which were validated by ANOVA, allowing the construction of the response surfaces (Figure 2). Figure 2d shows a region of lower a*_w_* at high ST concentrations (> 26.7 g/L) and intermediate GA (6.8 to 16.8 g/L), a pattern also observed for moisture (Figure 2f). This behavior may be related to the ability of ST to reduce free water mobility within the encapsulating matrix, resulting in lower a*_w_* values (Jafari et al. 2008). Conversely, the trials with the highest a*_w_* values (0.219 and 0.223) showed lower drying yields and EE, suggesting that higher residual water content may compromise powder stability (Cano‐Chauca et al. 2005).

The lowest moisture contents were recorded in formulations with high corn starch concentrations (> 26.7 g/L) and intermediate GA levels (6.8 to 16.8 g/L), reinforcing the role of ST in reducing the moisture content of the dried material, likely due to its hygroscopic structure and water‐retention capacity (Mastromatteo et al. 2011). Additionally, GA when used in higher amounts (> 26.7 g/L), may act as a plasticizer for ST, increasing its solubility and reducing crystallinity, thereby influencing moisture retention (Fonteles et al. 2012). Overall, the encapsulated materials showed low free water and moisture contents, minimizing undesirable interactions with other compounds and reducing the risk of oxidation or deterioration, which indicates good stability of the microcapsules.

### 3.2 Morphological and Structural Characteristics

**FIGURE 3:**
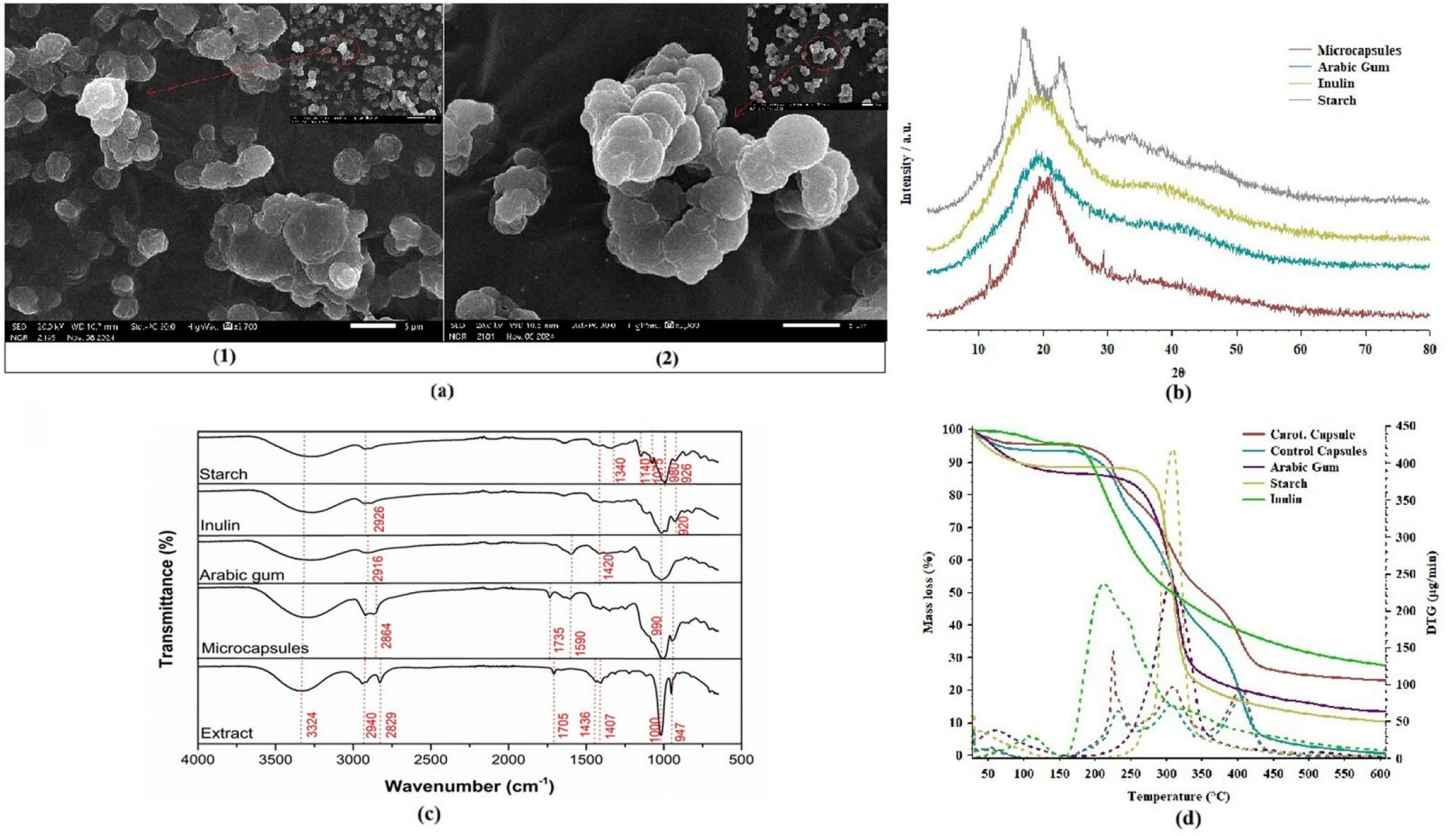
Structural, morphological, molecular, and thermal characterization of the wall materials and microcapsules. (a) SEM micrographs (10 and 5 µm scale bars) reveal predominantly spherical particles, with smoother surfaces in GA–rich formulations and more irregular textures when starch is present, indicating differences in film‐forming capacity. (b) X‐ray diffractograms show the typical amorphous profile of spray‐dried systems, with minor crystalline contributions from starch‐containing samples. (c) FTIR spectra display characteristic absorption bands associated with polysaccharides, and shifts in peak positions indicate interactions between the encapsulated carotenoids and the wall materials. (d) Thermograms demonstrate distinct degradation and thermal transition events, where gum arabic–based matrices exhibit higher thermal stability, while starch‐containing samples show earlier mass loss associated with polymer breakdown.

Figure 3a shows the SEM micrographs of the microcapsules obtained under the optimized conditions of 2^3^ CCRD and of the control sample (wall material only). The microcapsules containing 20% (v/v) carotenoid extract, 2 % (w/v) Tween 80, and 5 % of wall material GA:IN:ST (1:1:1, w/w/w) exhibited particles with an average size of 2.98 ± 0.76 µm, whereas the control microparticles (without extract), were significantly larger, with an average size of 4.13 ± 1.30 µm.

The microcapsules containing carotenoid extract displayed irregular morphology, with rough surfaces, agglomeration of smaller particles, and a less uniform distribution. In contrast, the control microparticles exhibited a predominantly spherical shape, with smoother surfaces and a more homogeneous distribution. These differences suggest that the presence of carotenoids interferes with the microencapsulation process, affecting both structural formation and final particle size. The possible interaction between carotenoids and the wall materials may hinder droplet coalescence during drying, leading to smaller particles that are more prone to agglomeration.

Differences in size and morphology directly affect properties such as appearance, surface area, reactivity, rehydration capacity, and solubility. These parameters are influenced by several factors, including drying conditions, wall material characteristics, solution viscosity, and solute concentration (Corrêa‐Filho et al. 2019). The smaller particles in the experimental group, with a larger surface area, may exhibit higher reactivity. The observed morphological differences can be attributed to the interactions between carotenoids and wall polymers. The presence of polar groups in carotenoids also favors hydrogen bonding with wall polymers, contributing to a more complex network and increased agglomeration.

Spray drying can lead to the formation of different surface structures. Recent studies have reported the occurrence of cavities on ST, based particles, primarily attributed to rapid solvent evaporation and crust formation during drying (Griep et al. 2023; Ding et al. 2022). The influence of wall material properties on particle morphology has also been highlighted, in the literature, with more rigid or structured matrices influencing particle shape, surface smoothness, and agglomeration tendencies (Wilkowska et al. 2017). Additionally, GA based systems have been shown to exhibit surface depressions and voids formed as a result of rapid heat and mass transfer during atomization (Corrêa‐Filho et al., 2019). This phenomenon is associated with rapid moisture loss, leading to wall solidification, particle shrinkage, and the possible release of trapped air. Therefore, the selection of wall materials combined with appropriate control of processing conditions is crucial for tailoring microcapsule morphology and optimizing their functional properties.

The X‐ray diffraction patterns (Figure 3b) reveal that both the microcapsules and the wall materials exhibit peaks at 2θ angles around 15°, 20°, and 30°, corresponding to reflections characteristic of wall materials with an amorphous structure, as evidenced by the broad diffraction peaks. This behavior is typical of carbohydrate, rich systems (Feng et al. 2020; Labuschagne 2018). In general, amorphous solids tend to show higher solubility and hygroscopicity compared with crystalline materials (Kaul et al. 2022; Tang et al. 2022).

The similarity of diffraction peaks suggests the presence of comparable crystalline planes, possibly related to the arrangement of polysaccharide chains (Jiang et al. 2021). Moreover, the diffraction pattern of carotenoid, containing microcapsules resembled that of the wall materials, indicating that the encapsulation process did not cause significant structural modifications to the particles. This suggests that the technique preserves the physicochemical properties of the encapsulated components, which may be advantageous for the stability and functionality of bioactive compounds (Patel et al. 2022).

The FTIR spectra (Figure 3c) of the wall materials, carotenoid extract, and microcapsules were obtained under the same acquisition conditions and are presented on a normalized and comparable scale within the range of 4000–650 cm^−^^1^, allowing a standardized evaluation of the main functional groups. The broad absorption band around 3300 cm^−^^1^ is attributed to O–H stretching vibrations characteristic of polysaccharides (GA, IN, and ST) and hydrogen bonding interactions within the matrix (Wang et al. 2021).

In ST characteristic carbohydrate bands were observed, including the signal at 2926 cm^−1^ corresponding to C–H stretching of methyl and methylenic groups, and the bands at 1420 and 1340 cm^−1^ associated with C–H deformation. Additional peaks at 1140, 1075, 980, and 926 cm^−1^ were attributed to C–O and C–OH linkages, typical of amylose and amylopectin structures (Quadrado and Fajardo 2020; Amaral et al. 2016; Lima et al. 2012).

The GA spectrum exhibited a typical polysaccharide profile, with a band near 2916 cm^−1^ attributed to C–H stretching vibrations. Absorption bands at 1590 and 1418 cm^−^^1^ were associated with asymmetric and symmetric stretching of carboxylate groups, while peaks at 1420, 1367, and 1340 cm^−1^ were related to C–H deformation and C–O–C stretching vibrations. Additionally, the broadband observed in the region of 1015–900 cm^−1^ further confirmed the presence of C–O–C and C–OH bonds, characteristic of the polymeric saccharide structure of GA (Al‐Maqtari et al. 2021; Chew et al. 2018).

Similarly, IN presented characteristic carbohydrate absorption bands, including a peak at 2926 cm^−1^ corresponding to C–H stretching vibrations, as well as bands at 1420–1410, 1330, 1215, 1108, 990, and 920 cm^−1^, which are associated with C–H bending, C–O–C stretching, and C–OH vibrations, confirming its polysaccharide nature (Akram and Garud 2020).

The carotenoid extract exhibited a broad band at 3324 cm^−1^, attributed to O–H stretching vibrations, possibly related to residual moisture or hydroxyl‐containing compounds. Absorption bands observed at 2940 and 2829 cm^−1^ correspond to C–H stretching vibrations of aliphatic chains, which are characteristic of hydrocarbons and fatty acid moieties commonly associated with carotenoid structures. A distinct peak at 1705 cm^−1^ was assigned to C = O stretching vibrations, indicating the presence of carbonyl groups, such as those found in oxygenated carotenoids. Peaks at 1436 and 1407 cm^−1^ were attributed to C–H bending (deformation) vibrations of methyl and methylene groups. Additionally, bands at 1000 and 947 cm^−1^ were associated with out‐of‐plane C–H bending vibrations, which are indicative of conjugated double bonds, a defining feature of the polyene chain in carotenoid molecules (Britton et al. 2004).

The microcapsules exhibited combined spectral features of both the wall materials and the carotenoid extract. Absorption bands observed at 2940–2920 cm^−1^ and 2870–2864 cm^−1^ were associated with aliphatic C–H stretching vibrations, originating from the polysaccharide matrix and the lipidic components of the carotenoids. Additional bands at 1735 cm^−1^ and 1604–1590 cm^−1^ were attributed to carbonyl (C = O) stretching vibrations, possibly related to ester or ketone functional groups and/or mild oxidative processes. The band at 990 cm^−1^, along with the peak at 947 cm^−1^, was assigned to out‐of‐plane C–H bending vibrations, characteristic of conjugated double bonds present in the carotenoid polyene chain. The overall similarity among the spectra indicates that the encapsulation process preserved the main functional groups of both the wall materials and the carotenoid extract. However, slight shifts in band positions and changes in intensity suggest the occurrence of hydrogen bonding interactions between the hydroxyl groups of the polysaccharide matrix and the carotenoid molecules. These interactions may contribute to the improved chemical stability of the encapsulated system, as previously reported by Souza et al. (2024).

### 3.3 Physical and Thermal Characteristics

The thermal conductivity of the microcapsules (under the optimized 2^3^ CCDR conditions) was 0.03 W/m C, a relatively low value, which indicates that the material has a limited capacity for heat conduction. This result suggests that the encapsulating matrix (composed of GA, IN, and ST) forms an effective thermal barrier, thereby reducing heat transfer and consequently protecting the bioactive compounds against thermal degradation. Previous studies have shown that encapsulating materials such as GA and ST generally exhibit low thermal conductivity, which is desirable for the protection of heat‐sensitive ingredients (Hirashima et al. 2012; Mothé and Rao 2000).

Thermal resistivity, the inverse of thermal conductivity, showed a high value of 34.93 m/C W, reinforcing the material's ability to resist heat transfer. High resistivity values are desirable in encapsulating materials, as they delay the thermal degradation of active compounds. According to Fang et al. (2019), microcapsules containing bioactive compounds with high thermal resistivity exhibit greater stability when exposed to elevated temperatures.

The thermal diffusivity of the microcapsules was 0.24 mm^2^/s, indicating that heat propagates at a moderate rate through the material. This value may be related to the nature of the polymers used in the formulation, which influences the rate of heat propagation within the microcapsules. Comparable values of thermal diffusivity were reported by Medina‐Jaramillo and López‐Córdoba (2025) when encapsulating lipophilic compounds in polymeric matrices containing GA and MD. Moderate thermal diffusivity can be beneficial, as it prevents a rapid increase in the internal temperature of the particle, reducing the risk of thermal degradation of bioactive compounds during processing and storage.

The bulk density of the encapsulated material was 0.43 g/mL, higher than the 0.29 g/mL reported by Zhao et al. (2019) for astaxanthin microparticles encapsulated with MD and gelatin by spray drying at 180°C. Bulk density is an important factor for the storage and transport of the material, as it affects the required packaging volume and may influence particle dispersibility in liquid or solid matrices (Correâ‐Filho et al. 2019). Higher densities may indicate better particle compaction, which can be advantageous for formulations requiring greater stability and reduced sedimentation in liquid systems.

The wall materials (GA, ST, and IN) exhibited distinct thermal degradation profiles in their thermograms (Figure 3d). Corn starch (ST) displayed an endothermic transition around 190°C, indicative of thermal degradation. Starches with higher amylose content exhibit limited molecular mobility, requiring elevated temperatures to break existing bonds and establish new ones with water (Weber et al. 2009). Gum arabic (GA) (red line) exhibited a significant endothermic peak near 140°C, which indicates a phase transition associated with dehydration (Hindi et al. 2017). Additionally, a more pronounced endothermic event around 190°C can be associated with polymer degradation. Inulin (IN) (black line) showed a significant endothermic event near 230°C, which is associated with thermal degradation caused by the rupture of chemical bonds in its polymeric structure. Compared with other polysaccharides, IN demonstrates greater thermal resistance prior to decomposition (Qiao et al. 2022).

For the microcapsule analysis, the green line represents the wall‐material microcapsules (without extract), while the gray line corresponds to the carotenoid‐containing microcapsules. The presence of carotenoids appears to interfere with the matrix, possibly reducing the transition temperature. The curve of the extract‐containing microcapsules shows greater heat absorption at lower temperatures, which may be explained by the effect of carotenoids on the glassy structure of the capsules, increasing the mobility of water molecules. The onset temperature of thermal degradation of the microcapsules, determined from the TGA curves, occurred at approximately 215°C (Coronel‐Aguilera and San Martín‐González 2015; Hoyos‐Leyva et al. 2018). This temperature corresponds to the beginning of significant mass loss and does not represent the maximum degradation peak (DTG).

The free carotenoid extract exhibited thermal transitions at lower temperatures compared to the encapsulated system. The degradation‐related events of the free extract occurred at temperatures below those observed for the microcapsules, indicating a positive temperature shift (ΔT) after encapsulation. The degradation onset of the encapsulated system (∼215°C) was higher than that observed for the non‐encapsulated extract, confirming improved thermal resistance.

Figure 3d shows the DSC analysis of the wall materials (GA, ST, and IN) and the microcapsules (with and without carotenoid extract). The thermal curve of corn starch (ST, blue dotted line) displayed an endothermic transition between 100°C and 150°C, possibly related to moisture loss or gelatinization, as also reported by Hirashima et al. (2012). Gum arabic (GA, red solid line) exhibited a pronounced endothermic event near 175°C, indicating the onset of thermal degradation, consistent with previous reports of GA decomposition within this temperature range (Mothé and Rao 2000). Inulin (IN, green dashed line) exhibited an endothermic transition around 225°C, suggesting thermal degradation or caramelization reactions, as discussed by Fang et al. (2019).

The microparticles without carotenoid extract (black dashed line) showed endothermic events near 175 and 225°C, reinforcing the influence of biopolymers on the system's thermal stability. In contrast, the microparticles containing carotenoids (black solid line) exhibited shifts in thermal transitions, suggesting that the extract interacts with the encapsulating materials, resulting in a measurable shift in degradation onset temperature (ΔT), indicating improved thermal resistance compared to the free extract. This behavior is consistent with the findings of do Amaral Souza et al. (2019), who reported an enhanced thermal stability of bioactive compounds when encapsulated in polymeric matrices. Furthermore, small exothermic events above 250°C may be related to carotenoid degradation, as previously reported for this class of compounds (Zhao et al. 2019).

Overall, the results indicate that microencapsulation contributed to the thermal protection of the extract, delaying its degradation and providing greater thermal stability. The presence of biopolymers in the encapsulating matrix acted as an effective thermal barrier, which is advantageous for applications in food, cosmetics, and pharmaceutical products requiring moderate thermal resistance. These findings reinforce the potential of the developed formulation for industrial applications involving heating or exposure to elevated temperatures.

The results summarized in Table 5 collectively confirm that the optimized microcapsule formulation produces a structurally stable and thermally resistant powder suitable for practical application. The combination of GA, IN, and ST yields microcapsules with moderate particle size and polydispersity, which is commonly associated with complex polysaccharide matrices and contributes to the balance between flowability and surface protection of encapsulated compounds.

**TABLE 5:** Physicochemical, structural, and thermal properties of the optimized microcapsule formulation obtained under the selected spray‐drying conditions (20 % extract, 2 % Tween 80, 5 % total wall material; GA:IN:ST ratio 1:1:1 (w/w/w); 120°C inlet temperature; 5.83 mL/min feed rate).

Parameter	Value*	Method/comment
Encapsulation efficiency (EE, %)	75 ± 1.2	As determined for the optimized formulation
Process yield (EY, %)	85 ± 0.84	Based on mass balance after spray drying
Load (µg_carotenoid_/g_capsule_)	20.5 ± 1.02	Measured amount of carotenoids removed from the capsule
Mean particle diameter (µm)	2.98 ± 0.76	SEM image analysis (*n* = 100 particles)
Size distribution	Polydisperse	Qualitatively observed by SEM histogram
Residual moisture (%)	5.5 ± 0.17	Gravimetric determination
Water activity (a*_w_*)	0.126 ± 0.09	Hygrometric measurement
XRD profile	Amorphous pattern with peaks at 2θ ≈	Typical of polysaccharide‐based wall materials
FTIR characteristic bands	2920 and 2870 cm^−^^1^	Aliphatic chains from carotenoids and polysaccharide matrix
Thermal conductivity (W/m°C)	0.03 ± 0.001	Measured by thermal properties analyzer
Thermal resistivity (m/°C W)	34.93 ± 1.7	Inverse of thermal conductivity
Thermal diffusivity (mm^2^/s)	0.24 ± 0.01	Determined experimentally
Thermal degradation onset (°C)	215	Observed in thermal analysis (TGA/DSC)
Bulk density (g/mL)	0.43 ± 0.02	Measured by mass per volume ratio

*Means ± standard error (*n* = 3).

The low moisture content and reduced a*_w_* indicate adequate drying efficiency and suggest good storage stability, as values in this range minimize agglomeration and inhibit microbial growth. The amorphous XRD pattern is consistent with expectations for spray‐dried polysaccharides, which favor the physical entrapment of bioactive components and have been associated with improved solubility and rehydration behavior.

In addition to these structural and physicochemical properties, the microcapsules exhibited a carotenoid loading of 20.5 µg per gram of capsules, which demonstrates that the matrix was able not only to encapsulate but also to retain a significant amount of bioactive compounds. The loading capacity (20.5 µg/g) may be considered moderate; however, this formulation strategy prioritized encapsulation efficiency and structural stability over maximum core loading. In practical food applications, carotenoids are typically incorporated at microgram‐per‐gram levels for fortification and functional purposes. Therefore, the obtained concentration is compatible with food system applications, where protection and controlled release are often more critical than high loading alone.

Thermal analyses revealed a degradation onset near 215°C, indicating that the matrix is thermally robust, which is an important feature when considering potential food or feed processing steps involving moderate heat. In addition, the low thermal conductivity and diffusivity suggest that the powder does not rapidly transfer heat, potentially further protecting the carotenoid‐rich core against thermal degradation.

Altogether, the physicochemical, structural, and loading attributes support the suitability of the optimized formulation for applications that require stability against moisture and heat, as well as resistance to structural collapse during processing and storage, reinforcing the effectiveness of the chosen wall material blend and processing conditions.

Although thermal stability was assessed through TGA and DSC analyses, oxidative, photo‐induced, and long‐term storage stability were not evaluated in this study. Considering that carotenoid degradation is strongly influenced by oxidation and light exposure, future investigations should address these parameters to further validate the technological applicability of the optimized ternary encapsulation system.

## 4 Conclusions

This study produced spray‐dried microcapsules containing carotenoid rich extracts from *S. salmonicolor* for potential use in food applications. The use of a mixed polysaccharide matrix (GA, IN, and ST) resulted in microcapsules with desirable technological attributes, including a predominantly amorphous structure, suitable morphology, and satisfactory EE. Structural analyses indicated interactions between the extract and the wall materials, contributing to the formation of a cohesive matrix.

Although oxidative or photo‐induced stability was not evaluated, the powdered microcapsules improved handling and facilitated their incorporation into formulated systems. Implications of this work include the development of a viable carrier system for carotenoids and a foundation for their controlled incorporation into food products. Future studies should assess their stability and release behavior under processing and storage conditions. The present findings demonstrate a promising platform for the industrial utilization of microbial carotenoids.

## Author Contributions

**Patrícia Griep**: conceptualization, investigation, funding acquisition, writing – original draft. **Rosicler Colet**: methodology, validation, visualization. **Elton Franceschi**: formal analysis. **Clarice Steffens**: formal analysis, writing – review and editing. **Jamile Zeni**: validation, visualization, writing – review and editing, supervision. **Eunice Valduga**: supervision, data curation, project administration, visualization, writing – review and editing.

## Funding

This study was financed in part by the Coordenação de Aperfeiçoamento de Pessoal de Nível Superior (CAPES).

## Ethical Statement

The authors have nothing to report.

## Conflicts of Interest

The authors declare no conflicts of interest.

## Data Availability

The datasets generated for this study are available on request to the corresponding author
